# Optimization of configuration parameters in a newly developed digital breast tomosynthesis system

**DOI:** 10.1093/jrr/rrt130

**Published:** 2013-12-01

**Authors:** Hye-Suk Park, Ye-Seul Kim, Hee-Joung Kim, Young-Wook Choi, Jae-Gu Choi

**Affiliations:** 1 Department of Radiological Science and Research Institute of Health Science, Yonsei University, #137 Backun Hall, 234 Maeji, Heungup, Wonju, Gangwon 220-710, South Korea; 2 Korea Electrotechnology Research Institute (KERI), Ansan, Geongki 426-170, South Korea

**Keywords:** digital breast tomosynthesis, microcalcification, projection view distribution, angular dose distribution

## Abstract

The purpose of the present work was to investigate the effects of variable projection-view (PV) and angular dose (AD) distributions on the reconstructed image quality for improving microcalcification detection. The PV densities at central and peripheral sites were varied through the distribution of 21 PVs acquired over ±25° angular range. To vary the AD distribution, 7 PVs in the central region were targeted with two, four and six times the peripheral dose, and the number of central PVs receiving four times the peripheral dose was increased from 3 to 11. The contrast-to-noise ratio (CNR) for in-focus plane quality and the full width at half maximum (FWHM) of artifact spread function (ASF) for resolution in the ***z***-direction were used. Although the ASF improved with increasing PV densities at two peripheral sites, the CNRs were inferior to those obtained with other subsets. With increasing PV density in the central area, the vertical resolution decreased but the CNR increased. Although increasing the central PV or AD concentrations improved image quality, excessive central densities reduced image quality by increasing noise in peripheral views.

## INTRODUCTION

The conventional mammogram used in breast cancer screening and diagnosis [1] provides high sensitivity and specificity for the early detection of non-palpable lesions associated with breast cancer. Full field digital mammography (FFDM) may improve image quality through advances in flat panel detector technology and image processing techniques [2, 3]. New flat panel detectors offer extremely high quantum efficiency and high resolution. However, both film and screen mammography and FFDM provide only 2D imaging; and in the projection of 2D images from 3D anatomical structures, overlapping structures produce shadows and artifacts. This leads to a high false positive rate [4] and an excess of mammography recalls and biopsies, with non-trivial morbidity risks [5].

Digital breast tomosynthesis (DBT) is an emerging 3D imaging technique that may resolve some limitations of conventional mammography. In DBT, a form of limited-angle cone-beam computed tomography (CT) [6, 7], a restricted number of 2D projections are acquired within a limited angle. This is generally accomplished by moving the X-ray tube along an arc in the conventional mammography projection geometry. The 3D volume of the breast is reconstructed from 2D projections using various reconstruction algorithms. Since DBT is adapted for angular undersampling, insufficient for 3D reconstruction of the breast, efforts to optimize reconstruction methods, detector performance, acquisition geometry and acquisition parameters have multiplied. Wu *et al.* developed a parallel reconstruction method based on a maximum-likelihood expectation maximization (MLEM) iterative algorithm [8], and also compared reconstruction algorithms [9]. Bissonnette *et al.* described a prototype DBT system using an amorphous selenium (a-Se) flat-panel detector [10]. Zhao *et al.* studied the detector performance of an a-Se detector with a pixel size of 85 µm and found great potential for a-Se detectors in DBT systems [11]. The geometrical parameters of image acquisition in DBT, including the number of projection views (PVs) and the total angular range, are not accessible in conventional mammography, and therein resides the power of DBT. The work of Sechopoulos *et al.* shows that maximizing the total angular range increases the vertical resolution, and increasing the number of PVs improves the contrast-to-noise ratio (CNR) for soft tissue lesions; specifically, an acquisition set of 13 projections over a 60º angular range gave the best image quality [12]. Based on observer performance, Chawla *et al.* found that increasing the angular range and signal size increases the detection rate. In that experimental context, acquisition of 15–17 projections over a 45º angular dose (AD) range gave the best performance [13, 14]. These studies were based on the uniform distribution of both PV and AD. In this work, we investigated the effects of various PV and AD distributions on the quality of reconstructed images obtained using a constant total dose. To analyze the dependence of image quality on these factors, we used the CNR and the artifact spread function (ASF) to quantify the in-focus plane artifacts along the *z*-axis. To verify our results qualitatively, we imaged the commercial breast-mimicking phantom BR3D (Model 020; CIRS, USA). Findings from this study may be applied to optimize acquisition configuration parameters in a DBT system.

## MATERIALS AND METHODS

### Description of the prototype DBT system

**Fig. 1.**
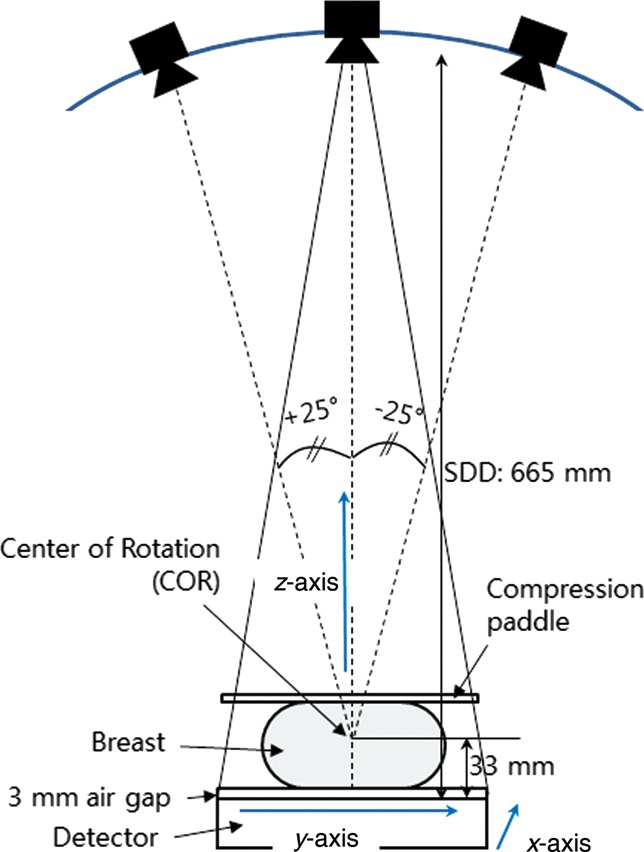
Schematic diagram showing the geometry of the prototype digital breast tomosynthesis (DBT) system: The *z*-axis extends outward, perpendicular to the chest, and the *y*-axis is the X-ray tube travel direction around the center of rotation (COR).

A prototype DBT system for breast imaging research (Korea Electrotechnology Research Institute [KERI], Changwon, Republic of Korea) was used in this study. The system has a CsI(Tl) scintillator/CMOS flat panel digital detector (2923MAM, Dexela Ltd, UK) with a pixel size of 0.0748 × 0.0748 mm^2^ and raw image data consisted of 14 bits. The actual detector area is 291 × 230 mm^2^, resulting in a matrix size of 3072 × 3888. The system can interchangeably provide tungsten/rhodium (W/Rh) (XM1016T, IAE, Italy) and molybdenum/molybdenum (Mo/Mo) (M-113SP, Varian Medical Systems, USA) anode/filter combinations. Image data presented here were taken with the W/Rh combination. The system uses a step and shoot mode to acquire projection images and the digital detector is stationary during image acquisition. During a single acquisition, the system synchronizes multiple X-ray pulses generated from the high frequency generator (MXR35, DRGEM, Korea) with the detector read/integrate cycle and X-ray tube motion. The X-ray tube voltage setting ranges from 20–40 kVp and the tube current–exposure time product value ranges from 50–150 mAs. No antiscatter grid was used. The imaging geometry of this DBT system is illustrated in Fig. 1. During a typical DBT scan, the X-ray tube travels in an arc of ±25º about a center of rotation (COR) located 33 mm above the detector surface. The source-to-detector distance (SDD) is 665 mm and the source-to-COR distance is 632 mm. For the geometrical calibration, the system provides angular information for each projection. From these data, the focal position of each single view was determined under the assumption that the focal point moves in an arc around the COR in a plane that intersects the detector plane at the chest wall side. The appropriate accuracy of this geometric model was validated by imaging an acrylic phantom with embedded metal balls after precise measurements for the angle of the system components were taken with a high-precision inclinometer (T233, Sherborne Sensors Ltd, UK).

### Image acquisition protocol

A tissue-equivalent phantom for mammography (Model 011A, CIRS, USA) was used for quantitative analysis, and a commercial breast-mimicking phantom (Model 020, CIRS, USA), designed to assess detectability of lesions of various sizes within a complex background that is generated by a heterogeneous tissue-equivalent material, was imaged to qualitatively verify our results. For each scan, the phantom images were acquired with an exposure technique of 30 kVp and the mAs values were adjusted until the intended mean glandular dose (MGD) to the phantom was achieved. The total MGD for all projection sets was estimated to be 2.0 mGy as a limiting factor. The MGDs were estimated using the method described by Sechopoulos *et al.* [15] as follows:

(1) $$D_g = X \times D_g N_0 \times N_\alpha \times \mu _{RGD} , $$

where *D*_*g*_ is the total MGD and *X* is the exposure measured at a specific point. At this specific point, the central ray meets the breast support plate. *D*_*g*_*N*_0_ is the normalized glandular dose for the zero degree projection, *N*_α_ is the number of PVs, and μ_*RGD*_ is the mean relative glandular dose (RGD) over all angles. The tables and equations for conversion from the entrance dose in the cranio–caudal (CC) view imaged with the tungsten target were used to compute the MGD [16].

#### Different PV distribution

**Fig. 2.**
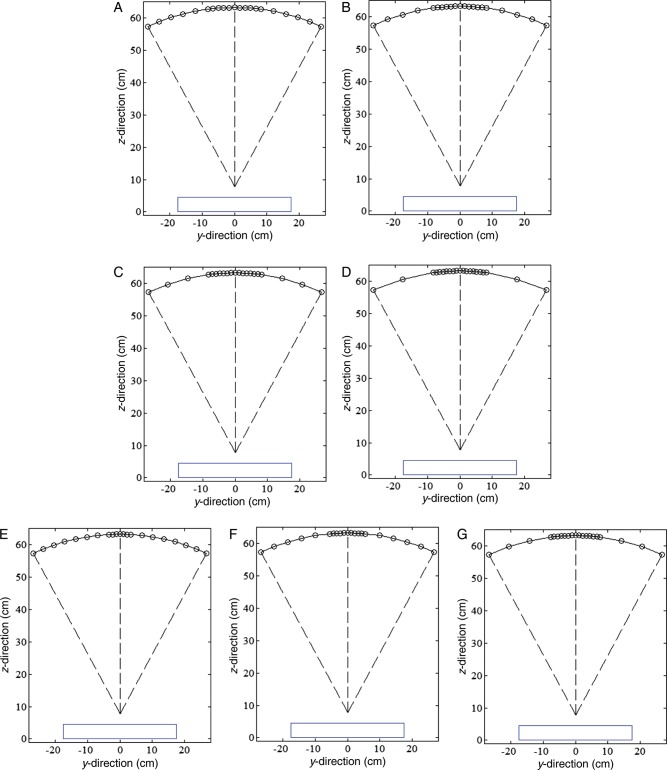
Seven subsets of PV density distribution at the central imaging site (PVc): of the set of 21 PVs, (**A**) 11, (**B**) 13, (**C**) 15, and (**D**) 17 were taken over ±7.5° angular range, and the remaining PVs, taken from ±7.5° to ±25°, were adjusted to have uniform angular spacing. Of the 21 PVs, (**E**) 7, (**F**) 11, and (**G**) 15 were taken at ±1° angular spacing increments from zero degrees in the central site. The blue line represents the detector.

**Fig. 3.**
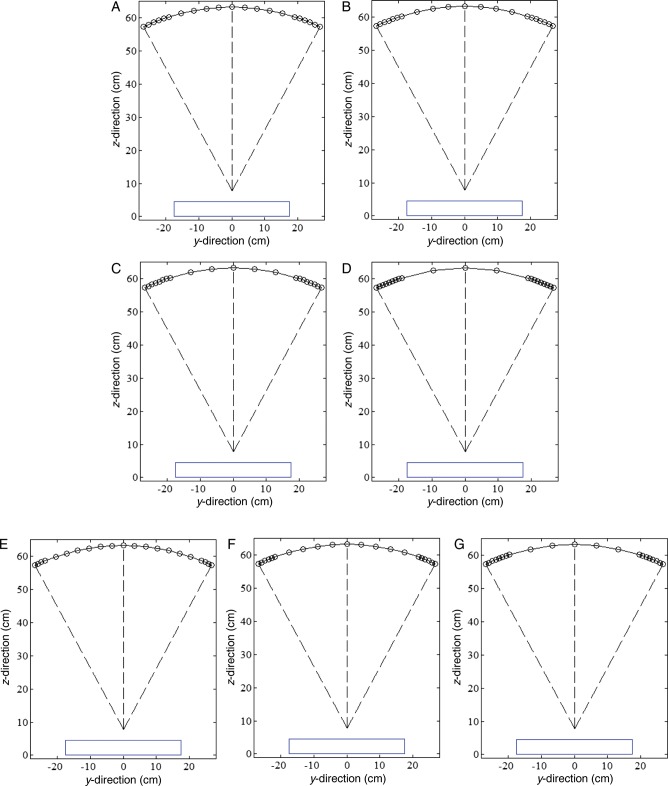
Seven subsets of PV density distribution at the peripheral imaging sites (PVp): in a set of 21 PVs, (**A**) 6, (**B**) 7, (**C**) 8, and (**D**) 9 were placed within the angular range of 7.5° at each peripheral site (i.e., from ±17.5° to ±25°). Of the 21 PVs, (**E**) 4, (**F**) 6, and (**G**) 8 were placed at ±1° angular spacing increments from the peripheral angular range limit (±25°), and the remaining PVs were adjusted to have uniform angular spacing across the angular range between the two peripheral sites.

To investigate the effects of variable PV distributions on reconstructed image quality, two general distributions were selected for a constant number of PVs acquired over ±25° angular range. All PV distributions were symmetric about the 0° PV, and the number of PVs in each distribution was chosen to be a constant of 21. In one distribution PVs were concentrated at the center of the scan (PV_c_), and in the other distribution PVs were concentrated at the peripheral sites (PV_p_). To evaluate the effect of the PV_c_, the number of PVs within ±7.5° angular range was increased from 11 to 17 PVs in increments of 2 PV, and the rest of the PVs, over ±7.5°– ± 25°, were adjusted for the same angular spacing. Images reconstructed from scans acquired with increasing the PV_c_ (i.e. with 7, 11 and 15 PVs at ±1° angular spacing within ±7.5° angular range) were evaluated. The seven subsets of the PV_c_ are shown schematically in Fig. 2. For PV_p_ distributions, 6, 7, 8 and 9 of the 21 PVs were placed within an angular range of 7.5° at each of the two peripheral sites (i.e. between ±17.5° and ±25°). For analysis, scans with 4, 6 and 8 of 21 PVs at ±1° angular spacing in each peripheral site were acquired, and the remaining PVs were spaced uniformly across the distance between the two peripheral sites. The seven subsets of the PV_p_ are shown schematically in Fig. 3. The same dose per projection was applied for all subsets and the MGD of each entire projection set was limited to 2.0 mGy.

#### Different AD distribution

To investigate the effects of AD distribution, 7 of the 21 PVs were placed at the center and targeted with four and six times the total peripheral dose (Table 1, No. 2, 3 and 4); and four times the total peripheral dose was delivered to 3, 7 and 11 of the 21 PVs placed at the center (Table 1, No. 5, 3 and 6). The AD distributions applied are shown in Table 1. All subsets presented in Table 1 were obtained with uniform PV distribution. Phantom imaging was repeated five times under the same imaging conditions. The same reconstruction method and detector were used for all imaging configurations.

**Table 1.** Angular dose distributions among projection views in DBT of a breast phantom

		Image weights							
No.	DBT acquisition configuration	0	±2.5	±5.0	±7.5	···	±12.5	···	±25
1	Uniform (reference)	1	1	1	1	···	1	···	1
2	2 × peripheral dose to 7 central PVs	2	2	2	2	···	0.5	···	0.5
3	4 × peripheral dose to 7 central PVs	2.4	2.4	2.4	2.4	···	0.3	···	0.3
4	6 × peripheral dose to 7 central PVs	2.57	2.57	2.57	2.57	···	0.21	···	0.21
5	4 × peripheral dose to 3 central PVs	5.6	5.6	0.23	0.23	···	0.23	···	0.23
6	4 × peripheral dose to 11 central PVs	1.53	1.53	1.53	1.53	···	1.53	···	0.42

All subsets were obtained with a uniform PV distribution. (There were 21 PVs over an angular range of ±25° with uniform ±2.5° angular spacing.)

### Reconstruction algorithms and image analysis

**Fig. 4.**
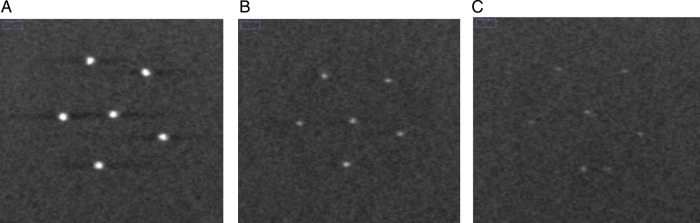
Reconstructed images of microcalcifications with nominal speck sizes of (**A**) 0.400, (**B**) 0.230, and (**C**) 0.165 mm in the tissue-equivalent phantom. The DBT slices were reconstructed from the reference scan that included 21 PVs acquired with uniform PV and AD distributions over ±25° angular range with ±2.5° angular spacing. Slices with objects in a focal plane are shown.

Reconstruction is the process by which data from the acquired projection views are used to create a 3D image. The reconstruction algorithm is an important element for optimization of the system, as it contributes to determining the overall image quality as well as the mechanical, geometric and dosimetric parameters. For this work, we applied the separable paraboloidal surrogates (SPS) algorithm to optimize microcalcification detection. The SPS algorithm is based on a penalized maximum likelihood (PML) reconstruction. As suggested by Fessler [17, 18], the algorithm was adapted using a surrogate function. The reconstructed volume was 20 cm × 14 cm × 5 cm, i.e. slightly larger than the phantom volume, and the voxel size was 0.075 mm × 0.075 mm × 1 mm (i.e. slice thickness was 1 mm). To quantitatively evaluate the quality of the image reconstructed in a focal plane, the CNR for microcalcifications in the reconstructed breast volume was computed for all projection sets. The CNR value is defined by [19]:

(2) $$CNR\left( z \right) = \displaystyle{{\overline I s\left( z \right) - \overline I _{BG} \left( z \right)} \over {\sigma _{BG} \left( z \right)}}, $$

where $\overline I_s$ and $\overline I_{BG}$ are the mean pixel values of the signal region of interest (ROI) and background ROI at a depth *z*, and *σ*_*BG*_ is the standard deviation of the pixel values in the same background ROI. The signal ROI was defined as a 4 × 4-pixel area in the center of the microcalcifications, and the background ROI was a chosen 50 × 50-pixel area far from all features. Three speck groups, with nominal sizes of 0.400, 0.230 and 0.165 mm, were analyzed as microcalcifications in the reconstructed tissue-equivalent phantom images (Fig. 4). The CNRs were obtained by averaging six microcalcifications in the speck groups and three repeated measurements. The error bars indicate the standard deviation of the measurements. To measure the quality of the vertical resolution of the reconstructed images and the artifact effect of features in the adjacent off-focus planes, the ASF of the microcalcifications were calculated as defined by Wu *et al.* [6, 9]:

(3) $$ASF\left( z \right) = \displaystyle{{\overline I s\left( z \right) - \overline I _{BG} \left( z \right)} \over {\overline I _s \left( {z_0 } \right) - \overline I _{BG} \left( {z_0 } \right)}}, $$

where *z*_0_ is the location of the in-focus plane of the feature and *z* is the location of the plane of interest. The full width at half maximum (FWHM) of the ASF was estimated from a polynomial function fitted to the ASF.

To optimize the configuration parameters, we employ the quality factor (QF) defined as [12]:

(4) $$QF = \displaystyle{{CNR} \over {FWHM\,of\,ASF}},$$

The QF combines the metrics for in-focus plane quality and for resolution in the *z* direction of the reconstructed images. The weights of these two metrics are equal in this computation of QF. Higher values of the QF correspond to configurations that deliver better image quality in terms of higher CNR and a lower FWHM of ASF. Thus the QF provides a criterion for selection among various sets of acquisition parameters.

## RESULTS

**Fig. 5.**
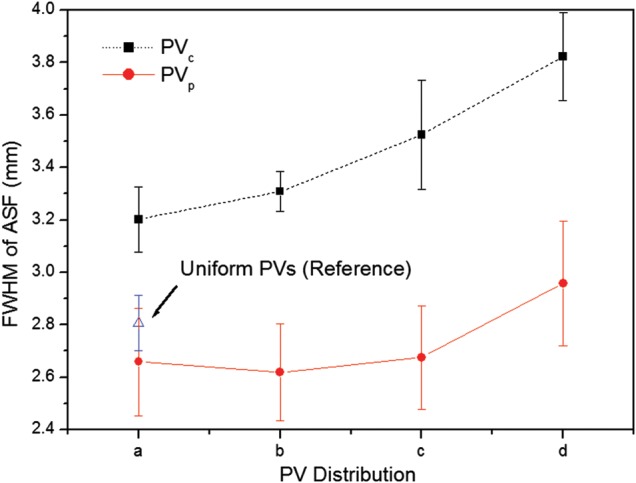
The FWHMs of ASFs in the *z*-axis averaged for six microcalcifications in the speck group of nominal size 0.400 mm. The eight subsets of parameters defining PV distributions were selected from (A), (B), (C), and (D) in Fig. 2 (PVc : dense PVs at central sites) and Fig. 3 (PVp : dense PVs at peripheral sites).

Figure 5 shows mean FWHMs of the ASF and their polynomial fits for different PV distributions. Eight subsets of the different PV distributions were selected from Fig. 2A–D (PV_c_) and Fig. 3A–D (PV_p_). As a reference for comparison, a set of 21 PVs acquired over ±25° angular range with uniform PV and AD distributions was also analyzed. For the PV_c_, the FWHM increased as the number of PVs increased. For the PV_p_, the subset with extremely dense distributions only increased, while the other subsets showed little difference. However, all subsets with PV_p_ yielded smaller FWHMs and thus had greater vertical resolution in the *z*-direction.

**Fig. 6.**
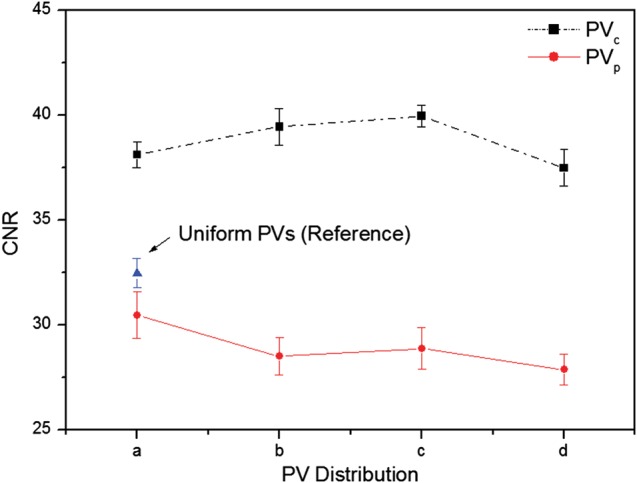
The CNRs for microcalcifications in the speck group of nominal size 0.400 mm. The eight subsets of parameters defining PV distributions were selected from (A), (B), (C), and (D) in Fig. 2 (PVc: dense PVs at central sites) and Fig. 3 (PVp: dense PVs at peripheral sites). The CNRs represent the average values for six microcalcifications in the speck group obtained in three repeated measurements. The error bars indicate the standard deviations of the measurements.

Figure 6 shows CNR results for microcalcifications in the speck group of nominal size 0.400 mm reconstructed from these eight subsets. All CNR results from the subsets with the PV_p_ were inferior to those from the subsets with the PV_c_. The reference set yielded a slightly higher CNR than the subsets with the PV_p_.

**Fig. 7.**
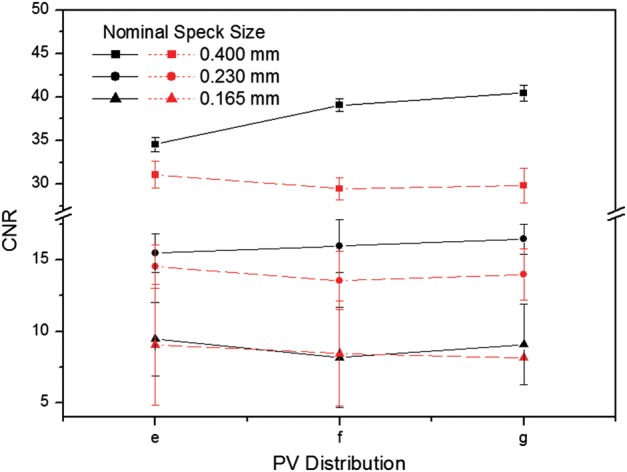
The CNRs for microcalcifications with nominal speck sizes of 0.400, 0.230 and 0.165 mm (shown in Fig. 4). The CNRs were obtained by averaging six microcalcifications in the speck group and three repeated measurements. The error bars indicate the standard deviation of the measurements. Six subsets of different PV distributions were selected from (E), (F) and (G) in Fig. 2 (PVc) and Fig. 3 (PVp). The solid and dashed lines are subsets in Fig. 2 and Fig 3, respectively.

The CNRs for six subsets are plotted in Fig. 7. The three speck groups with nominal speck sizes of 0.400, 0.230 and 0.165 mm, as shown in Fig. 4, were analyzed. The CNR of the six subsets increased with increasing speck size. The standard deviations of the CNR for the 0.400 mm speck size were relatively small compared with the smaller specks. The CNR gradually increased as the number of PVs increased for the PV_c_, while the CNR decreased for the PV_p_. It can be observed that microcalcifications with speck sizes of 0.400 and 0.230 mm have similar trends. However, for the smallest speck group, the CNRs did not show this consistent trend. The CNR from the PV_c_ yielded a higher CNR than that from the PV_p_, except for the smallest speck group.

**Fig. 8.**
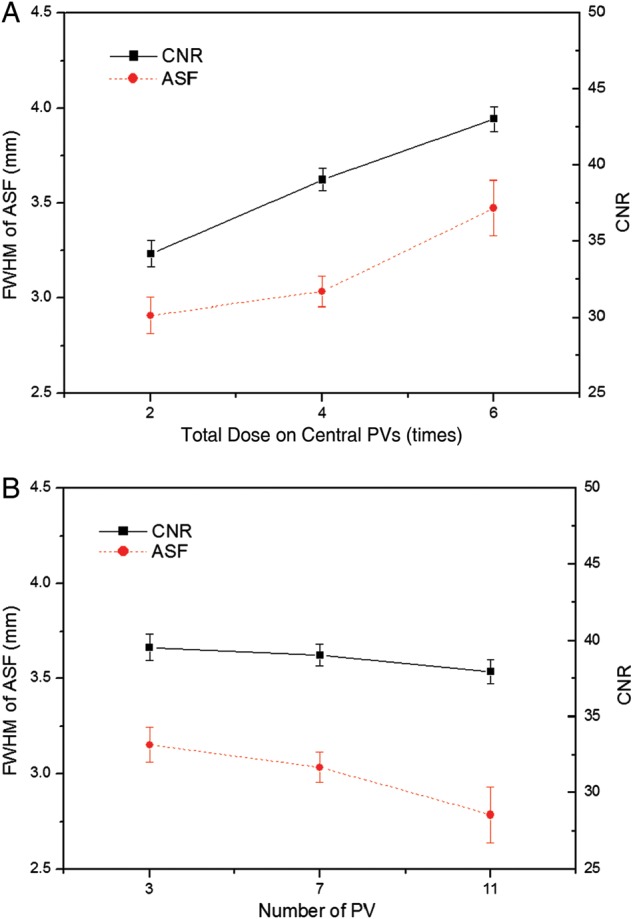
Comparison of the FWHMs and the CNRs using different AD distributions. (**A**) The total dose on the central 7 PVs was two, four and six times the peripheral dose. (**B**) The number of central PVs with four times the peripheral total dose was 3, 7 and 11 (shown in Table 1). The PV distribution in each AD distribution was fixed at 21 PVs over a ±25° angular range with ±2.5° spacing.

Figure 8 shows the FWHM and CNR for microcalcifications in reconstructions based on five different AD distributions (see in Table 1). Figure 8A shows that the CNR values improved as the total dose in the central seven PVs was increased, while the vertical resolution decreased. Corresponding plots are shown in Fig. 8B for distributions with 3, 7 and 11 of 21 PVs at the center receiving four times the peripheral dose. It was found that the CNR values decreased with increase in the number of PVs, while the vertical resolution was improved.

Table 2 shows results for quantitative analyses of DBT reconstructions with corresponding acquisition parameters. For comparison, the result of the reference scan that was acquired at 21 PVs over ±25° angular range with ±2.5° angular increment was included. For microcalcifications in the speck group of nominal size 0.400, the CNR increased with increasing PV and AD distributions at the center as compared with increasing PV_p_, while the subsets with PV_p_ generally yielded smaller FWHMs. However, subsets with PV_p_, such as Fig. 3D and G, gave greater FWHM than the reference with uniform PV distribution. The overall image quality factor, the acquisition configuration with PV_c_ or higher AD at the central region generally yielded higher QF than the reference configuration. In particular, the various AD distributions that concentrate the dose in the central views may yield the highest QF.

**Table 2.** The acquisition configuration and the results of the quantitative analysis performed on the DBT reconstructions

Effect	Group	DBT acquisition configuration	FWHM of ASF	CNR	QF
Reference	Uniform	21 PVs with ±2.5° angular spacing	2.807	32.465	11.566
PV distribution	PV_c_	Fig. 2A	3.202	38.125	11.906
		Fig. 2B	3.308	39.450	11.924
		Fig. 2C	3.525	39.965	11.338
		Fig. 2D	3.823	39.299	10.278
		Fig. 2E	2.923	34.560	11.822
		Fig. 2F	3.256	39.085	12.006
		Fig. 2G	3.502	40.470	11.555
	PV_p_	Fig. 3A	2.659	30.460	11.457
		Fig. 3B	2.619	28.490	10.878
		Fig. 3C	2.676	28.870	10.790
		Fig. 3D	2.958	27.857	9.418
		Fig. 3E	2.795	31.058	11.111
		Fig. 3F	2.777	29.478	10.617
		Fig. 3G	2.855	27.846	9.753
AD distribution	High dose in central site	No. 2 in Table 1	2.908	34.175	11.751
		No. 3 in Table 1	3.036	39.045	12.860
		No. 4 in Table 1	3.475	43.020	12.379
		No. 5 in Table 1	3.154	39.549	12.539
		No. 6 in Table 1	2.786	37.944	13.620

The 20 subsets were created to investigate the effects of different PV and AD distributions with a constant total dose on the reconstructed image quality. (The total MDG was 2.0 mGy.) For all acquisition configurations, microcalcifications in the speck group of nominal size 0.400 mm were used.

**Fig. 9.**
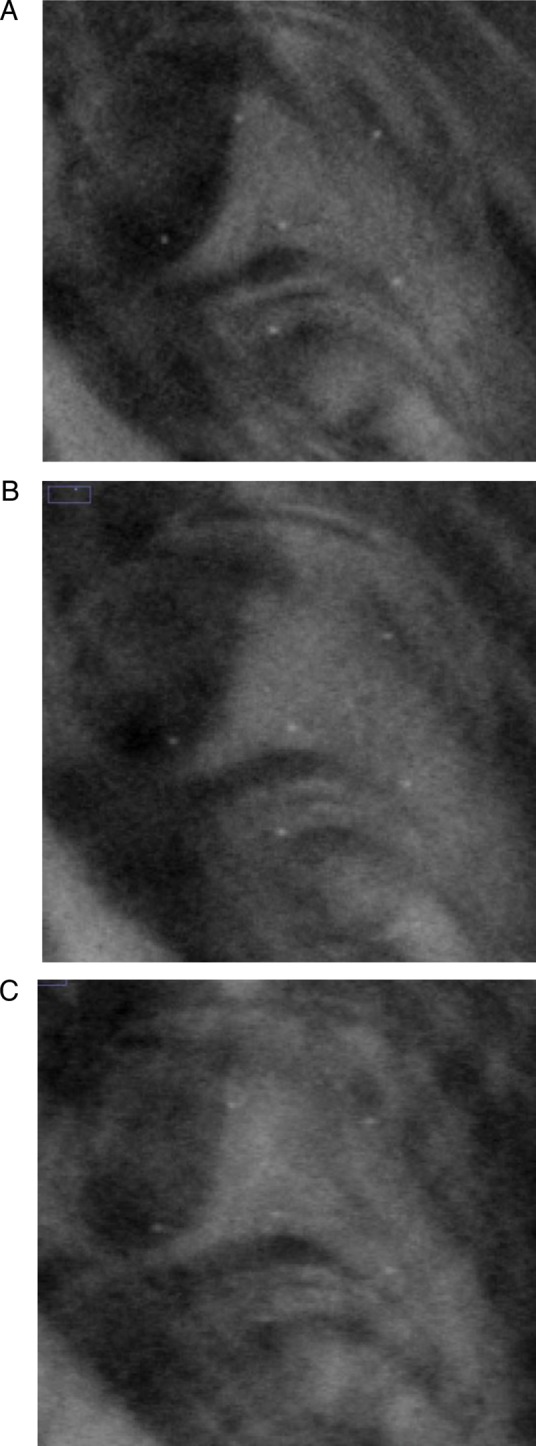
The in-focus DBT slices showing microcalcifications with nominal speck size of 0.165 mm in the commercial breast-mimicking phantom. The DBT slices were reconstructed using (**A**) PVc (Fig. 2B), (**B**) reference PVs, and (**C**) PVp (Fig. 3B).

**Fig. 10.**
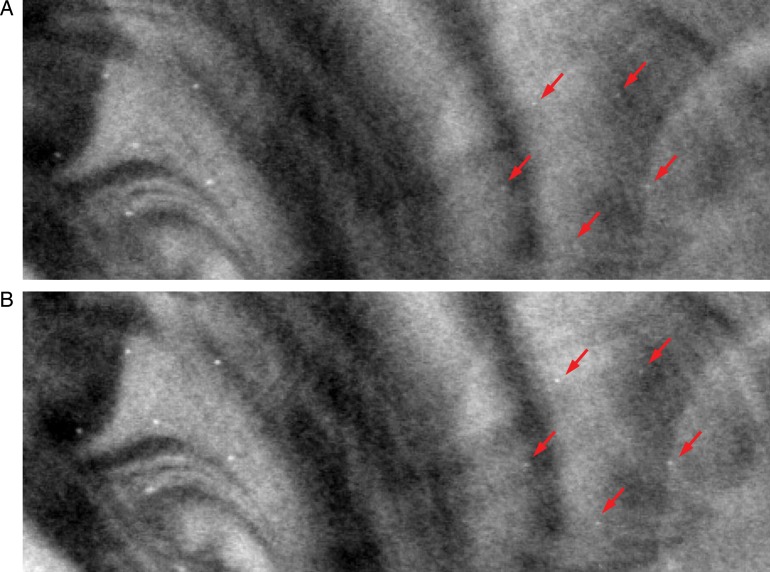
The in-focus DBT slices for microcalcifications with nominal speck sizes of 0.165 and 0.130 mm in the commercial breast-mimicking phantom. Example reconstructed images were acquired with 21 projection views over a ±25° angular range. (**A**) Uniform dose configuration (reference). (**B**) Non-uniform dose configuration (quarter of total dose is given for the central 11 PVs, and the rest of the dose is distributed over the remaining projection angles, as No. 6 in Table 1).

Figure 9 and 10 show in-focus slices revealing microcalcifications with nominal speck size 0.165 mm in the commercial breast-mimicking phantom. These reconstructed images of the phantom, shown in Fig. 9A, B and C, represent scans with high PV density in the central region (Fig. 2B), reference settings, and high PV densities at both peripheral sites (Fig. 3C), respectively. In Fig. 9C, the microcalcifications and breast parenchyma appear blurred. In the image shown in Fig. 9A, all six microcalcifications in the speck group can be observed with higher contrast. In comparison, the image in Fig. 9B is slightly more blurred than images from the subset with PV_c_.

To qualitatively verify the effect of varying the AD distribution, reconstructed images of the breast-mimicking phantom with the reference configuration and No. 6 in Table 1 are shown in Fig. 10. The different AD distributions with more dosage delivered to the central views of the tissue-equivalent phantom with the homogeneous background showed the highest QF (shown in Table 2), and improvement of image quality under the same AD distributions was also found for the commercial breast-mimicking phantom with the heterogeneous background, as shown in Fig. 10B. It can be seen that for uniform AD distributions, microcalcifications with nominal speck sizes of 0.130 mm appear to be blurred.

## DISCUSSION

In this study, we tested the effects of varying acquisition parameters, notably PV and AD distributions, on the quality of images reconstructed from data acquired with our prototype DBT system. With a tissue-equivalent breast phantom study, we compared the results of 20 subsets with 21 PVs over an angular range of ±25°, as shown in Table 2. The metrics (CNR, ASF and QF) that were used for quantitative evaluation can also be used to compare the results of other acquisition configurations for the DBT system. Our results demonstrate that for microcalcifications, non-uniform configurations with more PVs or dosage on the central site yielded higher image quality. In particular, different distributions for PVs and the AD (with the same total dose) have a strong impact on the vertical resolution in the *z*-direction. In varying the PV distribution, we found that PV_p_ yielded the greatest FWHMs, even slightly smaller than with the reference configuration. With increasing PV_c_ of the scan, the FWHMs declined, reflecting progressive decrease in data from peripheral sites. These findings concur with those in previous studies of extreme non-uniformity in density distribution at large angles [20]. Zhang *et al.* demonstrated that the PVs acquired close to the middle region contribute more to blurring in the out-of-focus planes than the peripheral PVs. The effects of PV density distribution on CNR in microcalcification images were similar to results previously obtained for relatively high-contrast microcalcifications [21]. In contrast to FWHM results, the CNRs increased slightly with increasing PV_c_, while CNRs decreased with increasing PV_p_. This may indicate that PV distributions with PV_p_ did not provide enough information to reconstruct contrast in the focal plane. As described by Sechopoulos *et al.*, the CNR of microcalcifications is sensitive to image noise, which increases with angular range [12]. With PV_p_, a greater proportion of PVs with large incidence angles contribute to image than with PV_c_, and noise is accordingly greater in the reconstructed images [22]. The CNR results from all subsets with PV_p_ were therefore inferior to results for other distribution subsets. The CNR values for all configuration subsets also depended on speck size. While CNRs for relatively high-contrast microcalcifications, e.g. those for speck sizes of 0.400 and 0.230 mm, followed similar trends, CNRs for the smallest speck group showed greater sensitivity to changing parameters. As shown in Fig. 7, the CNR of six subsets increased with increasing speck size because the larger microcalcifications produced relatively high contrast. Accordingly, the standard deviation in CNR was smaller for the larger speck groups than for the smallest speck group. We also investigated the effects of different AD distributions. Using the homogeneous tissue-equivalent phantom, we found that vertical resolution declined as the total dose delivered to 7 of 21 PVs placed at the center in the scan was increased, and also as the number of central PVs receiving four times the peripheral dose was decreased. As the AD delivered to the central views increased, noise increased at the two peripheral sites relative to contrast, because the dose per projection decreased. In this situation, the peripheral PVs gave insufficient information for image reconstruction in the *z*-direction. By similar reasoning, we explain the clear increase we observed in CNR values as dose increased in the central region of the scan. To optimize configuration parameters, we employed a QF that combines the metrics for in-focus plane quality and for resolution in the *z*-direction of the reconstructed images. Overall, the acquisition configurations with high PV_c_ or AD delivery at the central site yielded higher QF values than the reference configuration. In particular, increasing dose delivery to the central views may generate the highest QF. However, very high-dose concentrations toward the central views (see No. 18 in Table 2) may reduce the quality of the reconstructed image as increasing noise in peripheral views begins to dominate resolution in the *z*-direction. In addition to the tissue-equivalent phantom with homogeneous background, we used a commercial breast-mimicking phantom to reconstruct images acquired with all subsets of parameters. For the subsets with the PV_c_, all six microcalcifications in the speck group of nominal size 0.165 mm (relatively low-contrast microcalcifications) appeared with acceptably high contrast, while the subsets with PV_p_ generated blurred images of microcalcifications and breast parenchyma. For all subsets of AD distribution, all microcalcifications in the 0.165-mm speck group could be observed. The conspicuity of the microcalcification may be slightly greater when compared with the reference configuration. However, the uniform AD distribution produced a higher CNR. In this study, no subset of parameters gave results superior to the others in all metrics, indicating an inevitable trade-off between in-focus plane quality and resolution in the *z*-direction. In the design of a DBT system it is necessary to consider the trade-offs among these physical imaging properties. We have not shown these results to be valid for all specific types of lesion, breast backgrounds and image reconstruction methods, and for use of relative metrics such as the CNR and ASF; however, we have presented a general protocol for optimizing configuration parameters for a DBT system.

## CONCLUSION

The effects of variable PV and AD distributions on reconstructed image quality in DBT were investigated. Using a total glandular dose of 2.0 mGy to the imaged breast volume, twenty subsets of configuration parameters were created. Although increasing the central PV density or AD improved reconstructed image quality, an excessive PV density at the center increased noise in peripheral views to the detriment of image quality. Therefore, a strategic trade-off among physical imaging properties may be necessary to optimize diagnostic accuracy in a DBT system. Our results point to changes in analytical protocol that may improve image quality in DBT.

## FUNDING

This study was supported by the Korea Electrotechnology Research Institute (KERI) and by the National Research Foundation of Korea (NRF) grant funded by the Korean Government (MEST) (No. 2010-0018504).
